# Surgical myocardial revascularization outcomes in Kawasaki disease: systematic review and meta-analysis

**DOI:** 10.1515/med-2021-0242

**Published:** 2021-03-09

**Authors:** Antonio Salsano, Jingda Liao, Ambra Miette, Massimo Capoccia, Giovanni Mariscalco, Francesco Santini, Antonio F. Corno

**Affiliations:** Division of Cardiac Surgery, Ospedale Policlinico San Martino, University of Genoa, L.go Rosanna Benzi, 10, 16143, Genoa, Italy; Department of Integrated Surgical and Diagnostic Sciences (DISC), University of Genoa, Genoa, Italy; Cardiovascular Research Center, University of Leicester, Leicester, United Kingdom; Department of Intensive Care Medicine and Cardiac Surgery, Glenfield Hospital, University Hospitals of Leicester, Leicester, United Kingdom; Royal Brompton and Harefield NHS Foundation Trust, London, United Kingdom; Houston Children Heart Institute, Hermann Children’s Hospital, Houston, Texas, United States of America; University Texas Health, McGovern Medical School, Houston, Texas, United States of America

**Keywords:** aorto-coronary bypass grafting, coronary artery aneurisms, Kawasaki disease, surgical revascularization

## Abstract

**Background:**

Kawasaki disease (KD) is a systemic inflammatory condition occurring predominantly in children. Coronary artery bypass grafting (CABG) is performed in the presence of inflammation and aneurysms of the coronary arteries. The objectives of our study were to assess which CABG strategy provides better graft patency and early and long-term outcomes.

**Methods:**

A systematic review using Medline, Cochrane, and Scopus databases was performed in February 2020, incorporating a network meta-analysis, performed by random-effect model within a Bayesian framework, and pooled prevalence of adverse outcomes. Hazard ratios (HR) and corresponding 95% credible intervals (CI) were calculated by Markov chain Monte Carlo methods.

**Results:**

Among 581 published reports, 32 studies were selected, including 1,191 patients undergoing CABG for KD. Graft patency of internal thoracic arteries (ITAs), saphenous veins (SV), and other arteries (gastroepiploic artery and radial artery) was compared. ITAs demonstrated the best patency rates at long-term follow-up (HR 0.33, 95% CI: 0.17–0.66). Pooled prevalence of early mortality after CABG was 0.28% (95% CI: 0.00–0.73%, *I* ^2^ = 0%, tau^2^ = 0), with 63/1,108 and 56/1,108 patients, undergoing interventional procedures and surgical re-interventions during follow-up, respectively. Pooled prevalence was 3.97% (95% CI: 1.91–6.02%, *I* ^2^ = 60%, tau^2^ = 0.0008) for interventional procedures and 3.47% (95% CI: 2.26–4.68%, *I* ^2^ = 5%, tau^2^ <0.0001) for surgical re-interventions. Patients treated with arterial, venous, and mixed (arterial plus second venous graft) CABG were compared to assess long-term mortality. Mixed CABG (HR 0.03, 95% CI: 0.00–0.30) and arterial CABG (HR 0.13, 95% CI: 0.00–1.78) showed reduced long-term mortality compared with venous CABG.

**Conclusions:**

CABG in KD is a safe procedure. The use of arterial conduits provides better patency rates and lower mortality at long-term follow-up.

## 1 Introduction

Kawasaki disease (KD) is a systemic inflammatory condition occurring predominantly in children (80% of patients are younger than 5 years of age), first reported in 1974 as an acute febrile illness with mucocutaneous lesions and lymphadenopathy [1,2]. The incidence of KD varies across the world from 19 to 265 cases per 1,00,000 in children less than 5 years of age [2]. It can be complicated by a systemic vasculitis with particular involvement of the coronary arteries; if left untreated, 20% of children could develop coronary artery aneurysm [2]. In the acute phase of the disease, inflammatory formation of coronary aneurysms can occur, eventually associated with rupture of the latter, while thrombosis or narrowing of the affected coronary arteries can complicate the late phases [2,3,4,5,6]. In high income countries, KD remains the leading cause of acquired heart disease in children, with complication such as acute myocardial infarction [2,7,8,9]. Despite extraordinary results due to early recognition of the disease and treatment with intravenous administration of immunoglobulins [2,10], long-term studies showed that complications secondary to inflammation and aneurysmatic dilatation of the coronary arteries can still occur, with subsequent impairment of the left ventricular function [2,8,9,11,12,13,14]. Selective coronary angiography is indispensable in those situations to provide essential information for the decision making, before percutaneous or surgical myocardial revascularization [2,14,15,16,17]. Coronary artery bypass grafting (CABG) is performed in the presence of inflammation and aneurysms of the coronary arteries, using both arterial and/or venous grafts [17,18]. In the literature, only retrospective studies about CABG after KD are found and no meta-analysis has been published until now; grafts’ patency and long-term outcomes such as survival rates appear uncertain. Since the risk of progression of the systemic arteritis with formation of aneurysms in other arterial districts [19], such as internal thoracic artery (ITA) [20], the choice of the best type of surgical revascularization is still a matter of discussions [18].

The objectives of our study were to assess which surgical strategy of CABG provides better graft patency and early and long-term outcomes, through a literature systematic review and a Bayesian network meta-analysis.

## 2 Materials and methods

No ethical approval or review protocol was applicable for this study, since it was a review of existing literature. This work was in line with PRISMA (Preferred Reporting Items for Systematic Reviews and Meta-Analyses) guidelines (see Supplementary Materials – Appendix 1).

Primary endpoint is graft patency for ITAs, saphenous veins (SV), and other conduits (gastroepiploic [GEA] and radial [RA] arteries). Patency assessment at follow-up was performed using either invasive angiography or noninvasive computed tomographic angiography, and significative coronary stenosis (>70%) or occlusions were considered non-patent grafts [8].

Secondary endpoints were early mortality (in-hospital or 30-days mortality), need for interventional procedures and surgical re-interventions, and long-term mortality for arterial, venous, or mixed (arterial plus second venous graft) types of revascularization.

### 2.1 Search strategy

A systematic review using Medline, Cochrane, and Scopus databases has been performed in March 2020, incorporating a Bayesian network meta-analysis. A search strategy with a time interval from 1980 to 2020 was performed using the following search string for Medline (remaining strings have been reported in Supplementary Material): (cabg [Title/Abstract]) OR bypass [Title/Abstract] OR cardiac surgery [Title/Abstract] OR revascularisation [Title/Abstract] OR revascularization [Title/Abstract])) AND Kawasaki [Title/Abstract]. The articles were screened by three investigators (A. S., J. L., A. M.) initially by title and abstract, irrelevant articles were excluded, and subsequently full texts were evaluated for eligibility.

### 2.2 Inclusion and exclusion criteria

Inclusion criteria for the study were: (1) subjects involved in the study were patients with KD who underwent surgical revascularization; (2) comparison between different surgical strategies of myocardial revascularization; (3) at least one of the endpoints has to be included, such as graft patency, early and long-term mortality, and need of interventional procedures and/or surgical re-interventions; (4) sufficient quality data should have been provided in the original studies. Exclusion criteria for the study: (1) case reports, conference proceedings or reviews; (2) duplicates studies; (3) publications with full text in languages other than English. PICOS study design was used for inclusion/exclusion criteria (Table S1).

### 2.3 Data extraction

Three investigators (A. S., J. L., A. M.) independently identified and extracted all relevant data of eligible studies from original articles: first author’s name, year of publication, numbers of patients, mean age at surgery, gender, surgical strategy of myocardial revascularization, number of grafts, and endpoints such as graft patency, early and long-term mortality, and need of interventional procedures and/or surgical re-interventions. Data were retrieved only from the articles, and no attempt was made to get missing data from the authors. Any disagreement was solved by consensus.

### 2.4 Quality assessment

Study quality was assessed using the Newcastle-Ottawa Scale, a scale to evaluate the quality of non-randomized studies [21], and the US Preventive Services Task Force [22]. The Cochrane Risk of Bias tool was also used to evaluate the methodological quality of all included studies [23]. Quality assessment of each study is presented in Tables S2–S5.

### 2.5 Statistical analysis

Baseline and early mortality data were pooled using meta-analysis of means or proportions, with individual study effect size accounted for using inverse variance methods. Results were displayed as values and percentages with 95% confidence intervals (CI) or 95% credible intervals (CrI). Graft patency and long-term mortality for arterial, venous, or mixed CABG were analyzed across all arm-level studies, with direct and indirect comparisons using a mixed-treatment comparison based on a Bayesian hierarchical model. HRs and corresponding 95% credible intervals were calculated by Markov chain Monte Carlo methods using the “BUGSnet” package of R software (version 3.6.3; R Foundation, Vienna, Austria) [24]. Brooks–Gelman–Rubin plots method, trace plot, and density plot were used to assess the model convergence [25]. Besides, rank probabilities were calculated to obtain the hierarchical amount effects of multiple treatments. Given the graft type, CABG modalities, and time period over which included studies were conducted, a random-effect model was used. For the purposes of the mixed-treatment comparison, consistency in direct and indirect effects was assumed [26]. Heterogeneity between comparisons within the network was analyzed by examining *I* values for the random-effect model. Inconsistency was graphically examined using the BUGSnet nma.compare function to plot the individual data points’ posterior mean deviance contributions for the consistency model versus the inconsistency model [27]. For early mortality, need of interventional procedures, and surgical re-interventions, a frequentist approach (meta-analysis of proportions) has been applied. Pooled prevalence of adverse outcome has been calculated using the package “meta” of R software [28].

## 3 Results

### 3.1 Search results and study characteristics

The systematic literature review from 1981 to 2019 provided the following results: 581 reports and, after duplicates removal, 261 were screened. After screening of titles and abstract, 92 papers were excluded. The full texts of the remaining 169 articles were evaluated, and 137 studies were excluded with reasons. A total of 32 articles (observational studies and case series, no randomized controlled trial), including 1,191 patients, were selected [29,30,31,32,33,34,35,36,37,38,39,40,41,42,43,44,45,46,47,48,49,50,51,52,53,54,55,56,57,58,59,60].

Patients were mostly male with a mean age of 12.17 years and received CABG using 1.79 grafts per patient on average (Table 1).

**Table 1:** Pooled patients baseline data

Variables	Pooled data
Patients, *n*	1,191
Males, *n* (%)	718/965 (74.40%)
Age, mean (95% CI)	12.17 (10.67–13.88) years
Grafts	2,043
Grafts per patient, mean (95% CI)	1.79 (1.55–2.00)
Redo surgery, *n* (%)	56/1,114 (5.02%)
Off-pump surgery, *n* (%)	60/871 (6.89%)

**Figure 1:**
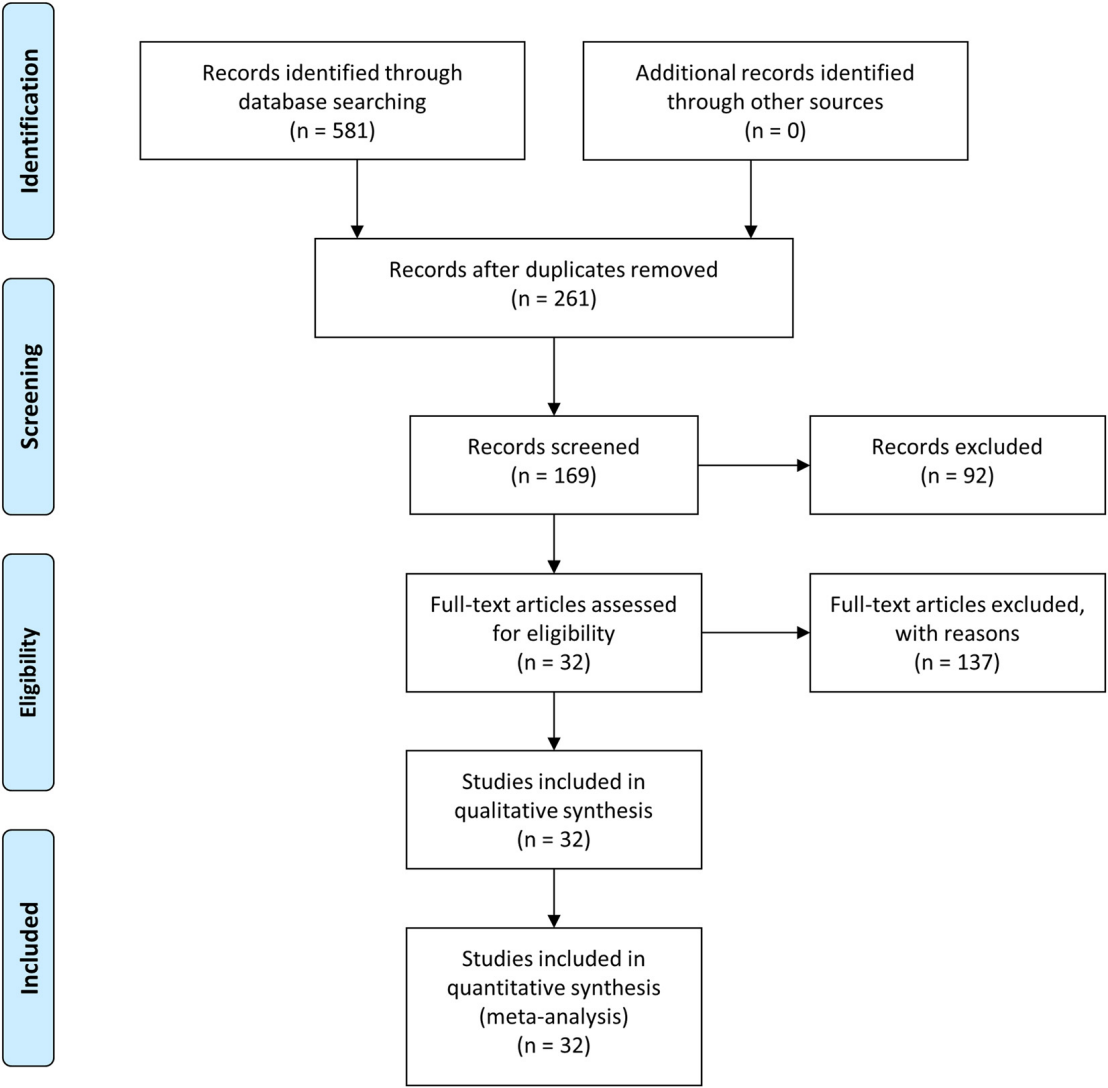
Flow diagram of the literature selection process.

A PRISMA flow diagram of the study selection process can be found in Figure 1. The quality assessment of the included studies is reported in Tables S2–S5. Detailed pooled patients’ data are presented in Table 1. For all studies, patients’ characteristics, main surgical strategies, and early outcome results are summarized in Table 2.

**Table 2:** Studies, patients’ characteristics, main surgical strategies, and extracted outcomes

Study	Year	Patients	Age (years)	Male sex	N. Graft	ITA	LITA	RITA	RA or GEA	SV	N. CABG	Arterial CABG	Venous CABG	Mixed CABG	Off-pump	On pump	Early patients mortality
Matsumoto et al. [29]	2019	3	21 ± 7.8	2 (67%)	3	3 (100%)	3 (100%)	0	0	0	3	3 (100%)	0	0	3 (100%)	0	0
Tadokoro et al. [30]	2019	92	14.9 ± 10.4	75 (82%)	175	125 (71%)	NA	NA	36 (21%)	14 (8%)	102	82 (80%)	8 (8%)	2 (2%)	17 (17%)	85 (83%)	0
Jeong et al. [31]	2018	20	20.15 ± 11.73	4 (20%)	44	40 (91%)	27 (61%)	13 (30%)	3 (7%)	1 (2%)	20	19 (95%)	0	1 (5%)	16 (80%)	4 (20%)	0
Beckmann et al. [32]	2017	2	27 ± 6	1 (50%)	6	4 (67%)	2 (33%)	2 (33%)	2 (33%)	0	2	2 (100%)	0	0	1 (50%)	1 (50%)	0
Ramírez-Marroquín et al. [33]	2017	7	6.14 ± 3.71	4 (57%)	13	12 (92%)	5 (38%)	7 (54%)	1 (8%)	0	7	7 (100%)	0	0	0	7 (100%)	0
Dionne et al. [34]	2017	11	8.3 ± 3.9	NA	NA	NA	NA	NA	NA	NA	11	7 (64%)	0	4 (36%)	NA	NA	0
Jang et al. [35]	2015	14	NA	NA	NA	11	NA	NA	2	1	14	12 (86%)	0	1 (7%)	NA	NA	1 (7%)
Tsuda et al. [36]	2014	90	NA	NA	155	NA	NA	NA	NA	NA	90	NA	NA	NA	NA	NA	0
Guo et al. [37]	2010	8	21.25 ± 13.57	5 (63%)	20	8 (40%)	6 (30%)	2 (10%)	1 (5%)	11 (55%)	8	3 (37.5%)	2 (25%)	3 (37.5%)	2 (25%)	6 (75%)	1 (13%)
Muta et al. [38]	2010	81	13 ± 9	60 (74%)	131	99 (76%)	NA	NA	20 (15%)	12 (9%)	81	69 (85%)	2 (4%)	9 (11%)	10 (12%)	71 (88%)	1 (1%)
Viola et al. [39]	2010	5	8.8 ± 3.63	NA	11	9 (82%)	5 (45.5%)	4 (36.5%)	0	2 (18%)	5	4 (80%)	0	1 (20%)	0	5 (100%)	0
Legendre et al. [40]	2010	2	0.94 ± 1.04	NA	4	4 (100%)	2 (50%)	2 (50%)	0	0	2	2 (100%)	0	0	NA	NA	0
Kitamura et al. [41]	2009	114	NA	86 (75%)	198	154 (78%)	111 (56%)	43 (22%)	14 (7%)	30 (15%)	114	90 (79%)	3 (3%)	21 (18%)	0	114 (100%)	0
Mueller et al. [42]	2009	2	NA	NA	5	3 (60%)	2 (40%)	1 (20%)	0	2 (40%)	2	1 (50%)	0	1 (50%)	NA	NA	0
Wakisaka et al. [43]	2009	13	10.38 ± 5.78	11 (85%)	32	11 (34%)	10 (31%)	1 (3%)	1 (3%)	20 (63%)	13	0	3 (23%)	10 (77%)	NA	NA	0
Kitamura et al. [44]	2008	2	25 ± 7	2 (100%)	4	3 (75%)	2 (50%)	1 (25%)	1 (25%)	0	2	2 (100%)	0	0	0	2 (100%)	0
Tsuda et al. [45]	2008	2	7.5 ± 3.53	0	2	2 (100%)	2 (100%)	0	0	0	2	2 (100%)	0	0	NA	NA	0
Tsuda et al. [46]	2007	67	NA	48 (72%)	125	95 (76%)	0	0	13 (10%)	17 (14%)	71	54 (76%)	0	17 (24%)	7 (10%)	64 (90%)	0
Tsuda et al. [47]	2004	244	13 ± 8	188 (77%)	435	310 (70%)	NA	NA	40 (10%)	85 (20%)	244	NA	NA	NA	4 (2%)	240 (98%)	1 (0%)
Yamauchi et al. [48]	2004	21	11.86 ± 7.42	17 (81%)	32	29 (91%)	26 (81%)	3 (9%)	3 (9%)	0	21	21 (100%)	0	0	0	21 (100%)	0
Inoue et al. [49]	2001	6	9.3 ± 2.25	5 (83%)	8	5 (62.5%)	4 (50%)	1 (12.5%)	0	3 (37.5)	6	3 (50%)	2 (33%)	1 (17%)	NA	NA	0
Suda et al. [50]	2000	2	8 ± 1.41	2 (100%)	2	0	0	0	0	2 (100%)	2	0	2 (100%)	0	0	2 (100%)	0
Yoshikawa et al. [51]	2000	100	10 ± 5	72 (72%)	168	138 (82%)	99 (59%)	39 (23%)	9 (5%)	21 (13%)	100	79 (79%)	2 (2%)	19 (19%)	NA	NA	0
Mavroudis et al. [52]	1999	4	6.12 ± 4.8	NA	5	5 (100%)	3 (60%)	2 (40%)	0	0	4	4 (100%)	0	0	0	4 (100%)	0
Kitamura et al. [53]	1994	168	10.6 ± 8.1	127 (76%)	288	143 (50%)	NA	NA	12 (4%)	133 (46%)	168	114 (68%)	0	54 (32%)	0	168 (100%)	1 (0%)
Suzuki et al. [54]	1990	26	NA	NA	37	28 (76%)	24 (65%)	4 (11%)	0	9 (24%)	26	NA	NA	6 (23%)	0	26 (100%)	1 (4%)
Kitamura et al. [55]	1983	5	12.2 ± 9.4	4 (80%)	9	0	0	0	0	9 (100%)	5	0	5 (100%)	0	0	5 (100%)	0
Suma et al. [56]	1981	8	7.75 ± 2.55	5 (63%)	13	0	0	0	0	13 (100%)	8	0	8 (100%)	0	NA	NA	0
Hirose et al. [57]	1986	5	NA	NA	10	2 (20%)	NA	NA	0	8 (80%)	5	NA	NA	NA	NA	NA	0
Torii et al. [58]	1996	9	NA	NA	18	9 (50%)	NA	NA	9 (50%)	0	9	9 (100%)	0	0	NA	NA	0
Ohara et al. [59]	1989	22	NA	NA	28	20 (71%)	NA	NA	0	8 (29%)	22	15 (68%)	2 (9%)	5 (23%)	NA	NA	0
Takeuchi et al. [60]	1992	36	NA	NA	62	27 (43.5%)	22 (35.5%)	5 (8%)	8 (13%)	27 (43.5%)	36	NA	NA	NA	NA	NA	0

N, number; CABG, coronary artery bypass graft; ITA, internal thoracic artery; LITA, left internal thoracic artery; NA, not applicable; RITA, right internal thoracic artery; RA, radial artery; GEA, gastroepiploic artery; SV, saphenous vein; PCI, percutaneous coronary intervention.

Categorical data were presented as frequencies and percentages, continuous variables as means, and standard deviation (if reported in the included studies).

### 3.2 Results from network meta-analysis

#### 3.2.1 Primary outcome: graft patency

Overall, 1,191 patients were included in the present meta-analysis, with an average of 1.79 (95% CI: 1.55–2.00) grafts per patient. ITA, RA or GEA, and SV conduits were used; however, the exact numbers of each graft type and graft target vessels were incompletely reported. Of the 32 studies, 15 arm-level papers were included to perform the Bayesian network meta-analysis, with a total of 1,441 grafts with 324 significative stenosis/occlusions [30,31,32,33,39,41,42,43,44,47,49,53,54,57,59]. Mean follow-up for coronary angiography across studies was 92.19 months (follow-up: min 3–max 264 months). For the primary outcome, we compared patency of ITAs, SV, and other arteries (GEA and RA) at follow-up. Patency rates at follow-up for ITAs, SV, and other arteries are 87.82 ± 12.41%, 65.98 ± 27.84%, and 77.63 ± 22.75%, respectively. The network model, trace plot, and density plot for graft patency are shown in Figures S1 and S2.

**Figure 2:**
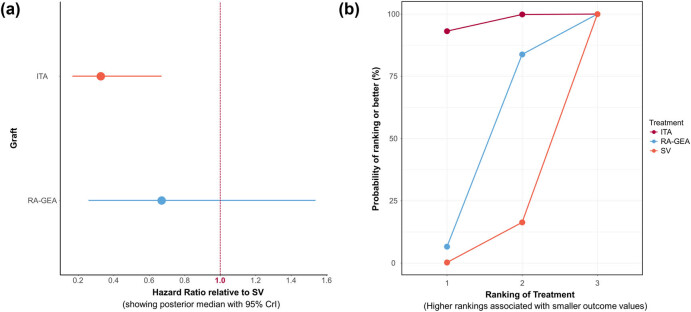
(a) Forest plot for graft patency. (b) Sucra plot for graft patency showing rank probability analysis.

Summary results for all grafts patency at follow-up are shown in Figure 2a, with SV used as reference. These pooled results show that ITAs and other arteries (GEA and RA) are superior to SV. Rank probability analysis for graft patency at follow-up demonstrates that ITA had higher probabilities of being the first most effective treatment (Figure 2b).

**Figure 3:**
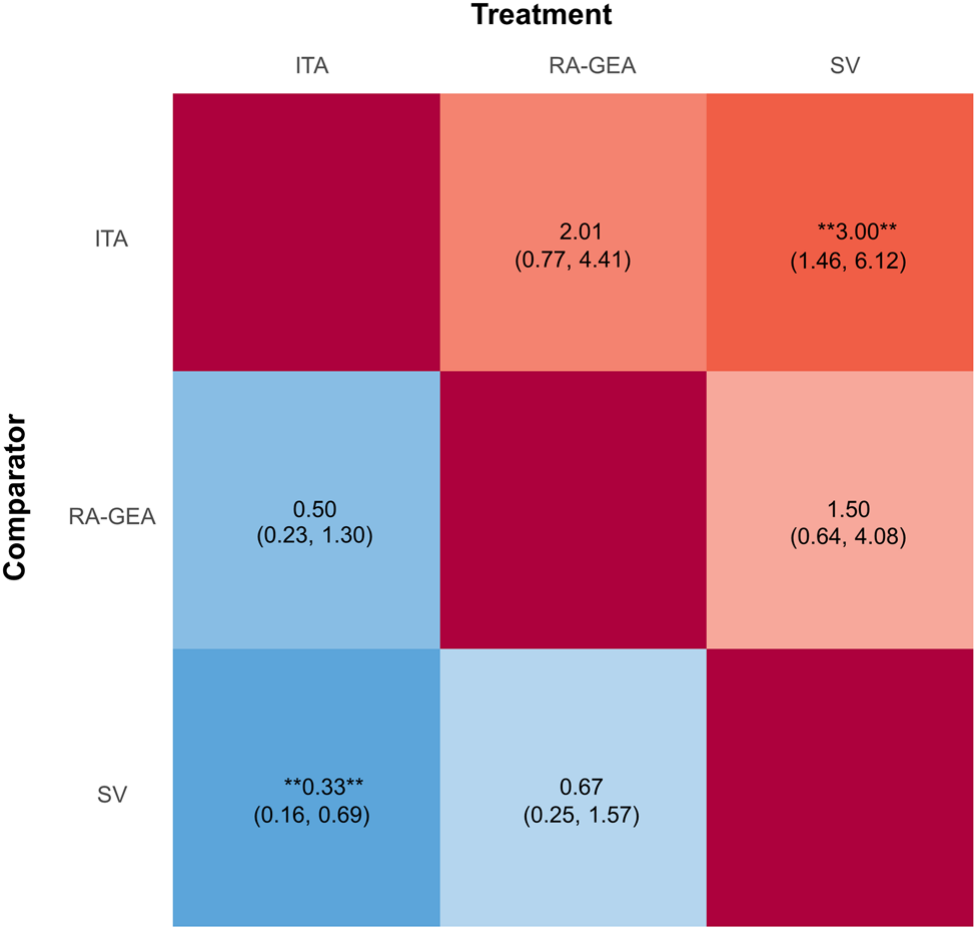
League table for graft patency.

The efficacy of different treatments in terms of graft patency at follow-up using HR and corresponding 95% CrI is displayed in Figure 3. Pairwise comparisons for graft patency at follow-up are shown in Figures S3a–c. *I* ^2^ demonstrated that heterogeneity was low for SV versus ITAs comparison and was moderate for SV and ITAs versus other arteries (GEA and RA). After comparison of consistency and inconsistency models through individual data points’ posterior mean deviance contributions, we conclude that there is a lack of evidence to suggest inconsistency within the network (Figure S4).

#### 3.2.2 Secondary outcomes: early and long-term mortality, need of interventional procedures, and surgical re-interventions

In all 32 studies included in the meta-analysis, only 6 early deaths were reported among 1,191 patients. Pooled prevalence of early mortality after CABG was 0.28% (95% CI: 0.00–0.73%, *I* ^2^ = 0%, tau^2^ = 0, Figure S5).

Twenty six studies reported interventional procedures and surgical re-interventions rates: 63/1,108 and 56/1,108 patients underwent interventional procedures and surgical re-interventions at follow-up (mean 110.35 months, 95% CI: 28.50–264.00 months), respectively.

Pooled prevalence of interventional procedures was 3.97% (95% CI: 1.91–6.02%, *I* ^2^ = 60%, tau^2^ = 0.0008, Figure S6). Across the studies that reported interventional procedures, high heterogeneity demonstrated elevated variability across the included studies.

Among the interventional procedures, percutaneous transluminal balloon angioplasty was performed only for graft stenosis, while percutaneous transluminal rotational ablation was done for coronary artery lesions. 7 DES stents were implanted for conduit stenoses (5 SVG graft, 1 ITA graft, and 1 RA graft). Pooled prevalence of surgical re-interventions was 3.47% (95% CI: 2.26–4.68%, *I* ^2^ = 5%, tau^2^ <0.0001, Figure S7).

**Figure 4:**
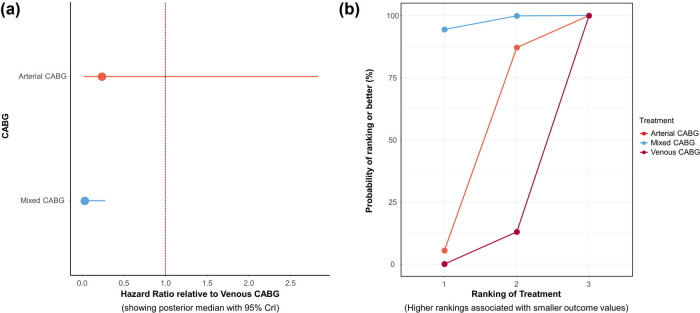
(a) Forest plot for long-term mortality compared to SV. (b) Sucra plot for long-term mortality showing rank probability analysis.

Patients treated with arterial, venous, and mixed (arterial plus second venous graft) CABG were compared to assess long-term mortality. Eight arm-level papers out of 32 studies were included to perform the network meta-analysis [31,34,39,41,42,43,49,53]. Mean follow-up across studies for long-term mortality was 142.56 months (follow-up: min 48–max 264 months). Survival at follow-up after arterial, venous, and mixed CABG is 99.07 ± 2.27%, 83.33 ± 28.87%, and 99.87 ± 0.33%, respectively. The network model, trace plot, and density plot for long-term mortality are shown in Figures S8 and S9. Summary results are shown in Figure 4a, while rank probability analysis is shown in Figure 4b.

The efficacy of different treatments using HR and corresponding 95% CrI is displayed in Figure S10. Pairwise comparisons at follow-up are shown in Figure S11a–c. There is a lack of evidence to suggest inconsistency within the network model (Figure S12). Briefly, mixed CABG (HR 0.03, 95% CrI: 0.00–0.30) and arterial CABG (HR 0.13, 95% CrI: 0.00–1.78) showed reduced long-term mortality compared with venous CABG.

More in deep, focusing on the comparison between long-term mortality for arterial CABG vs. mixed CABG, we are facing with 58 single CABG out of a total of 133 cases (43.6%) in which at least one arterial conduit has been employed (Figure S11c) [34,39,41,42,49]. It appears that the use of arterial conduit, even better if applied in a multiple CABG setting, provides benefits on long-term mortality. Although the low number of deaths at follow-up is a positive finding, caution is required in interpreting these results and it needs to be confirmed in future studies.

## 4 Conclusions

This network meta-analysis aimed to assess which CABG strategy provides better graft patency and long-term outcomes. Results from 32 studies with a total of 1,191 cases demonstrated that CABG for KD is a safe procedure with satisfying long-term outcomes.

Our systematic review and meta-analysis showed a low early mortality after CABG in KD, ranging between 0 and 1%. The excellent efficacy of surgical revascularization was also demonstrated by 10-year survival rates above 90%, with a 3.5–4% rate of interventional procedures or surgical re-interventions during the follow-up.

The choice of conduits in coronary artery surgery remains a debated and controversial issue, and the following points should be considered before CABG: the expected long-term patency of the graft, considering that the factors that may influence long-term patency are the presence of competitive flow from the native vessel or collaterals, further development of atherosclerosis, and abnormal coronary artery structure and function at the site of the anastomosis. The choice between arterial revascularization with ITA, free gastroepiploic artery, or free radial artery versus venous CABG with saphenous vein has to be well-discussed prior to surgery. The growth potential of the graft relative to the somatic growth of the patient, particularly in the pediatric age, has to be taken into account: ITA in situ appears to grow with the patient, but there are doubts on the growth of free grafts conduits. However, the risk of progression of the systemic arteritis with formation of aneurysms in other arterial districts, such as ITAs [19,20], has to be considered too.

Recently published guidelines recommended a tailored approach to individual practice [61,62]. The standard surgical strategy of myocardial revascularization used in adult patients was not been adequately studied in patients with KD, giving rise to numerous speculations. The use of bilateral thoracic artery was appealing in younger patients, although diabetes, obesity, chronic obstructive disease, and female sex remained adverse factors and should be taken into serious consideration even in adult patients. The radial artery represented a valid alternative to the saphenous vein with encouraging medium to long-term results. The right gastroepiploic and inferior epigastric arteries remain of limited application with less supporting evidence for their usage in the adults. Allografts and artificial grafts are very rarely, if ever, used. The choice of conduit should be addressed for each patient or group of patients and balanced on anatomical criteria, patient background, conduit availability, and surgical expertise [18,61,62].

In the future, we could assist in a rising number of KD cases due to the potential association with pediatric COVID-19 [63], for which it is even more actual and important to know the better strategy of treatment for coronary complications after KD.

To assess the latter, in this study we compared graft patency of ITA, SV, and other arteries (gastroepiploic artery and radial artery); and patients treated with arterial, venous, and mixed (arterial plus second venous graft) CABG to assess long-term mortality.

Our meta-analysis demonstrated that arterial conduits provided better patency rates at 10 years follow-up, with ITAs as the first most effective surgical option, when compared to SV. No progression of the systemic arteritis in other arterial districts was detected in the included studies.

Arterial or mixed (arterial plus a second venous graft) CABG, seen as a surrogate for the use of arterial conduits for revascularization, has been shown to be associated with higher patients’ survival rates. This result complies with the superiority of the arterial grafts when used for surgical myocardial revascularization for KD patients.

### 4.1 Limits of the study

We have identified the following limits. Network meta-analyses for early mortality, need of interventional procedures, and surgical re-interventions were not conducted. Randomized clinical trial evidences for graft patency following CABG after KD were not published. We pooled observational studies results on the topic, including not adjusted comparative studies. This is a potential source of underpowering that increases heterogeneity where there is variability between patency results. Moreover, a low-moderate risk of bias for the included studies should be taken into account in the interpretation of the results. Nevertheless, this systematic review and meta-analysis represents an overview of the surgical myocardial revascularization in KD and may represent a starting point for further studies and refinement of the technique.

Meta-regression has not been applied. It is unlikely that this represents a source of bias since young patients with few comorbidities have been included in this review. Given that, it cannot certainly be excluded since <5% of patients develop obstructive lesions resulting in ischemic coronary disease regardless of the administration of gamma-globulins [8]. Graft patency was compared regardless of the territory of revascularization. “Grey literature” was not investigated. Furthermore, as the included studies were published between 1981 and 2019, improvements in patency outcomes or long-term mortality could be expected to vary over time due to operative and therapeutic improvements.

In conclusion, our results demonstrate that CABG in KD is a safe procedure, with an overall early mortality rate of 0.28% and rates of surgical re-interventions and interventional procedures at follow-up of 3.47% and 3.97%, respectively. The use of arterial conduits was associated with better patency rates and lower mortality at follow-up.
